# Progress in Surgery of the Stomach

**Published:** 1904-01-16

**Authors:** 


### PROGRESS IN SURGERY OF THE STOMACH.

**Gastric Ulcer.**—The indications for operation in the hæmorrhages of gastric ulcer are considered by Mathieu and Roux[1] under three headings:—(1) Acute and profuse hæmorrhages not yielding to medical treatment; (2) small chronic hæmorrhages, insignificant singly, but repeating themselves incessantly in spite of strict medical treatment; (3) acute profuse hæmorrhages easily arrested by medical means, but repeating themselves at longer or shorter intervals, and leaving the patient enfeebled on each occasion. In the first class the great difficulty in the operation is to expose the bleeding point, and, since the vast majority of acute hæmorrhages can be arrested by medical means, these ought always to be resorted to in the first instance. When they fail, the general state of the patient is usually such that any severe operative procedure would destroy the last chance of recovery. In the second class, that of the recurrent slight hæmorrhage, the ulcer is almost always situated near the pylorus, causing some stenosis, and it is in this variety that surgery has given its best results. Gastro-enterostomy gives immediate relief both to the hæmorrhage and to the stenosis, and is strongly to be recommended. In the third group, of repeated profuse hæmorrhages, excision of the ulcer is the ideal treatment, if possible, failing which gastro-enterostomy is to be performed, especially if there is any associated stenosis. Moullin[2] considers that the ideal treatment of chronic ulcer is by excision. The wound caused in this way heals in a few days. The patient can be given liquid food in small quantities as soon as the vomiting due to the anæsthetic has ceased; and the scar left is a simple linear one, not likely to contract or to lead to the development of adhesions. The success of the operation, both as regards the actual rate of mortality and the completeness of recovery, is simply dependent upon the condition of the ulcer and the state to which the patient has been reduced at the time of the operation. These cases may be divided into two classes. There are, first, those in which the operation is performed deliberately, as a preventive measure, in intelligent anticipation of consequences, which are practically certain to follow. Secondly, there are those in which the operation is performed as a last chance, when course after course of medical treatment has been tried and failed, and when the patient is in the last stage of exhaustion, either from hæmorrhage or from prolonged starvation, or both. Mitchell[3] considers that the indications for treatment in gastric ulcer are as follows:—1. All cases in which the cause of the symptoms is mechanical are essentially surgical. By no possibility could medicinal or dietetic treatment be expected to cure either an hour-glass stomach or a case of pyloric stenosis. For dilated stomach treatment by washing out is a very favourite one and gives great relief at first by removing the toxic products of feeble digestion and excess of acids, but at the best it is only palliative; and while it may be recommended in the first instance, common sense suggests that the sooner the actual cause of the dilatation is removed the better, as the risk of operation increases in direct proportion to the delay. 2. There is, however, a very large proportion of cases in which no such definite indication exists. Here the question becomes one for the physician, and he is not justified in recommending operation until he has exhausted every other method of treatment. Before any operation is undertaken the patient should be submitted to a period of prolonged rest in bed (not less than one month) with rigid dieting or rectal feeding. If this fails to give relief operation is clearly indicated. On the other hand, it is obviously wrong to allow sufferers to struggle along year after year in the position of chronic invalids without at least offering them the chance of relief by surgical means. 3. Hæmatemesis sometimes calls for surgical aid, but the operative mortality is very high. A great difficulty lies in the surgeon's way in all these cases. He can rarely say definitely what he is going to do till the abdomen is opened. The operation, in this sense at least, must be regarded as exploratory, but with increased accuracy of diagnosis, and the knowledge gained by operative work, our opinion will gradually become more definite and precise. Moynihan[4] places great confidence in rigidity of the abdomen as a sign of perforation in gastric ulcer. The operation for perforated ulcer should be conducted speedily, and all means adopted to save the patient from shock. The excision of the ulcer is not necessary. If there is much soiling a free flushing of the cavity is necessary; if the operation is done within ten or twelve hours a gentle wiping of the surrounding area with wet swabs will suffice. Drainage, as a rule, is not necessary except in the late cases. When adopted it should be free, a split tube and a gauze wick being placed in the original incision and in a second suprapubic opening. In duodenal ulceration the perforation may be very large, and when the gap is stitched up a narrowing of the calibre of the duodenum results, and it may therefore be necessary to give an alternative route from the stomach by performing gastro-enterostomy. Carless says that where an ulcer has caused pyloric obstruction treatment may be directed either to the pylorus, being more or less radical, or one may have to depend on gastro-enterostomy. (1) Pyloroplasty is a simple procedure and should be a very successful operation. Well marked limitations, however, exist to the utility of this operation. In the first place, it can only be undertaken in the absence of adhesions. Secondly, it is very difficult, or almost impossible, to open out and to suture up a very thick and indurated pylorus. Thirdly, if the stomach is much dilated and sags downwards, the contents are likely to stagnate in the pouch thus formed and no improvement will follow. Again, when the motor power of the stomach is diminished pyloroplasty is of very little use. Finally, it must not be forgotten that occasionally duodenal ulcers co-exist with the gastric variety and cicatrisation of these may lead to duodenal stenosis when pyloroplasty would be useless. (2) Pylorectomy is sometimes valuable in the removal of chronic ulcers involving the pylorus which has become hard and indurated. (3) Gastro-plication is sometimes useful as an adjuvant to one or other of these proceedings. (4) Where, however, these proceedings directed towards the pylorus itself are for one reason or another contra-indicated gastro-enterostomy must be relied on and the result will probably be most beneficial.

[1] Med. Chron., July, 1903, p. 248. [2] Brit. Med. Jour., Oct. 17, 1903, p. 985. [3] Lancet, Aug. 29, 1903, p. 593. [4] Lancet, July 18, 1903, p. 149.

*(To be continued.)*

